# Struct2GO: protein function prediction based on graph pooling algorithm and AlphaFold2 structure information

**DOI:** 10.1093/bioinformatics/btad637

**Published:** 2023-10-17

**Authors:** Peishun Jiao, Beibei Wang, Xuan Wang, Bo Liu, Yadong Wang, Junyi Li

**Affiliations:** School of Computer Science and Technology, Harbin Institute of Technology (Shenzhen), Shenzhen, Guang Dong 518055, China; School of Computer Science and Technology, Harbin Institute of Technology (Shenzhen), Shenzhen, Guang Dong 518055, China; School of Computer Science and Technology, Harbin Institute of Technology (Shenzhen), Shenzhen, Guang Dong 518055, China; Guangdong Provincial Key Laboratory of Novel Security Intelligence Technologies, Harbin Institute of Technology (Shenzhen), Shenzhen, Guangdong 518055, China; Center for Bioinformatics, Faculty of Computing, Harbin Institute of Technology, Harbin, Heilongjiang 150001, China; Key Laboratory of Biological Bigdata, Ministry of Education, Harbin Institute of Technology, Harbin, Heilongjiang 150001, China; Center for Bioinformatics, Faculty of Computing, Harbin Institute of Technology, Harbin, Heilongjiang 150001, China; Key Laboratory of Biological Bigdata, Ministry of Education, Harbin Institute of Technology, Harbin, Heilongjiang 150001, China; School of Computer Science and Technology, Harbin Institute of Technology (Shenzhen), Shenzhen, Guang Dong 518055, China; Guangdong Provincial Key Laboratory of Novel Security Intelligence Technologies, Harbin Institute of Technology (Shenzhen), Shenzhen, Guangdong 518055, China; Key Laboratory of Biological Bigdata, Ministry of Education, Harbin Institute of Technology, Harbin, Heilongjiang 150001, China

## Abstract

**Motivation:**

In recent years, there has been a breakthrough in protein structure prediction, and the AlphaFold2 model of the DeepMind team has improved the accuracy of protein structure prediction to the atomic level. Currently, deep learning-based protein function prediction models usually extract features from protein sequences and combine them with protein–protein interaction networks to achieve good results. However, for newly sequenced proteins that are not in the protein–protein interaction network, such models cannot make effective predictions. To address this, this article proposes the Struct2GO model, which combines protein structure and sequence data to enhance the precision of protein function prediction and the generality of the model.

**Results:**

We obtain amino acid residue embeddings in protein structure through graph representation learning, utilize the graph pooling algorithm based on a self-attention mechanism to obtain the whole graph structure features, and fuse them with sequence features obtained from the protein language model. The results demonstrate that compared with the traditional protein sequence-based function prediction model, the Struct2GO model achieves better results.

**Availability and implementation:**

The data underlying this article are available at https://github.com/lyjps/Struct2GO.

## 1 Introduction

As the expression products of genes and macromolecules in organisms, proteins are the main material basis of life activities, widely existing in various cells, providing many functions such as catalysis, cell signal, and structural support, playing a key role in life activities and functional execution. At the same time, the study of proteins can better grasp life activities on a molecular level, which has important practical significance for the management of diseases, the creation of new medications and the improvement of crops. Because of the progressing high-throughput sequencing technology, protein sequence data are increasing exponentially. At present, more than 100 000 proteins have been obtained by biological experiments in the Universal Protein (UniProt) (UniProt Consortium 2018) database with standard functional annotations. This accounts for only 0.1% of the proteins in the UniProt database. However, the method of verifying protein functions based on biological experiments is time-consuming and labor-intensive and has strict requirements on equipment and funds, which cannot meet the increasing annotation demand, so it is necessary to design an efficient protein function prediction method.

The protein function prediction problem can be viewed as a multi-label binary classification problem, that is, by extracting the features of the given protein and mapping it to the space of protein function labels. A variety of data sources can be tapped to obtain protein function prediction features, such as protein sequence, protein structure, protein family, and protein–protein interaction network, etc. The most commonly used information source is protein sequence and interaction network. The protein function labels can be standardized through The Gene Ontology Consortium (2017), which is a database established by the Gene Ontology Consortium to define and describe genes and their products. According to different functional scopes, Gene Ontology includes three independent branches: Cellular Component, Molecular Function and Biological Process.

Generally, the study of protein function prediction can be separated into three stages. The initial step is the classic sequence-based method, such as BLAST (Altschul *et al.* 1990), which calculates the similarity between protein sequences and transfers annotations between proteins with similarity scores exceeding a certain threshold. This method has great limitations in the prediction of protein functions without sequence similarity. The second stage is the machine learning method based on a decision tree and support vector machine, of which the representative is the multi-source *k* nearest neighbors (Lan *et al.* 2013) algorithm, which integrates multiple similarity measurement methods to find the *k* nearest neighbors of the current protein, and the annotation of the current protein is determined by calculating the weighted average of the function of its neighboring proteins. In 2018, the DeepGO (Kulmanov *et al.* 2018) model proposed by Kulmanov *et al.* was the initial application of deep learning to protein function prediction, learning features from the protein sequence matrix through convolutional neural networks, and combining the embedding vectors of protein nodes in the PPI network for function prediction, and then enter the third phase of deep learning models. The following year, the team proposed the DeepGOPlus (Kulmanov and Hoehndorf 2020) model, which does not rely on the embedding vectors of protein nodes in the protein–protein interaction network, but captures sequence similarity information through the diamond (Buchfink *et al.* 2015) sequence alignment tool and combines CNN to extract sequence features to improve prediction performance. DeepGraphGO (You *et al.* 2021) leverages the family and domain information of the sequence to provide the nodes with initial characteristics and then utilizes graph convolutional networks to acquire the structural information of the PPI network. Building on this, PSPGO (Wu *et al.* 2022) proposed a multi-species label and feature propagation model based on a protein sequence similarity network and PPI network.

All of the above methods use protein sequence as the information source to predict GO terms, however, simply utilizing protein sequence information cannot reveal the correlation between protein functions. And models that obtain homologous sequence features based on the PSSM method exhibit lower sensitivity to single amino acid substitutions (Arya *et al.* 2022). Structure determines function is a universal rule in nature (Dawson *et al.* 2017, Mitchell *et al.* 2019). Hence, despite disparate sequences, two proteins with analogous structures may possess analogous functions (Brenner *et al.* 1996, Holm and Sander 1996, Krissinel 2007, Sebastian and Contreras-Moreira 2013). It is imperative to create techniques that utilize protein structural data to anticipate functions to compensate for the disparity between protein sequence and function. DeepFRI (Gligorijević *et al.* 2021) has demonstrated encouraging outcomes in the annotation of protein functions through the utilization of experimentally determined protein structural databases. Although only a limited number of proteins have experimental structures, AlphaFold2 (Jumper *et al.* 2021) has achieved a remarkable advancement in protein structure prediction, attaining an unprecedented level of accuracy in the prediction of protein structures at the atomic level, and in most cases has shown accuracy comparable to experiments, and has public released 214 million protein structure information, including humans, which will further promote the development of methods for predicting protein functions using structure.

**Figure 1.**
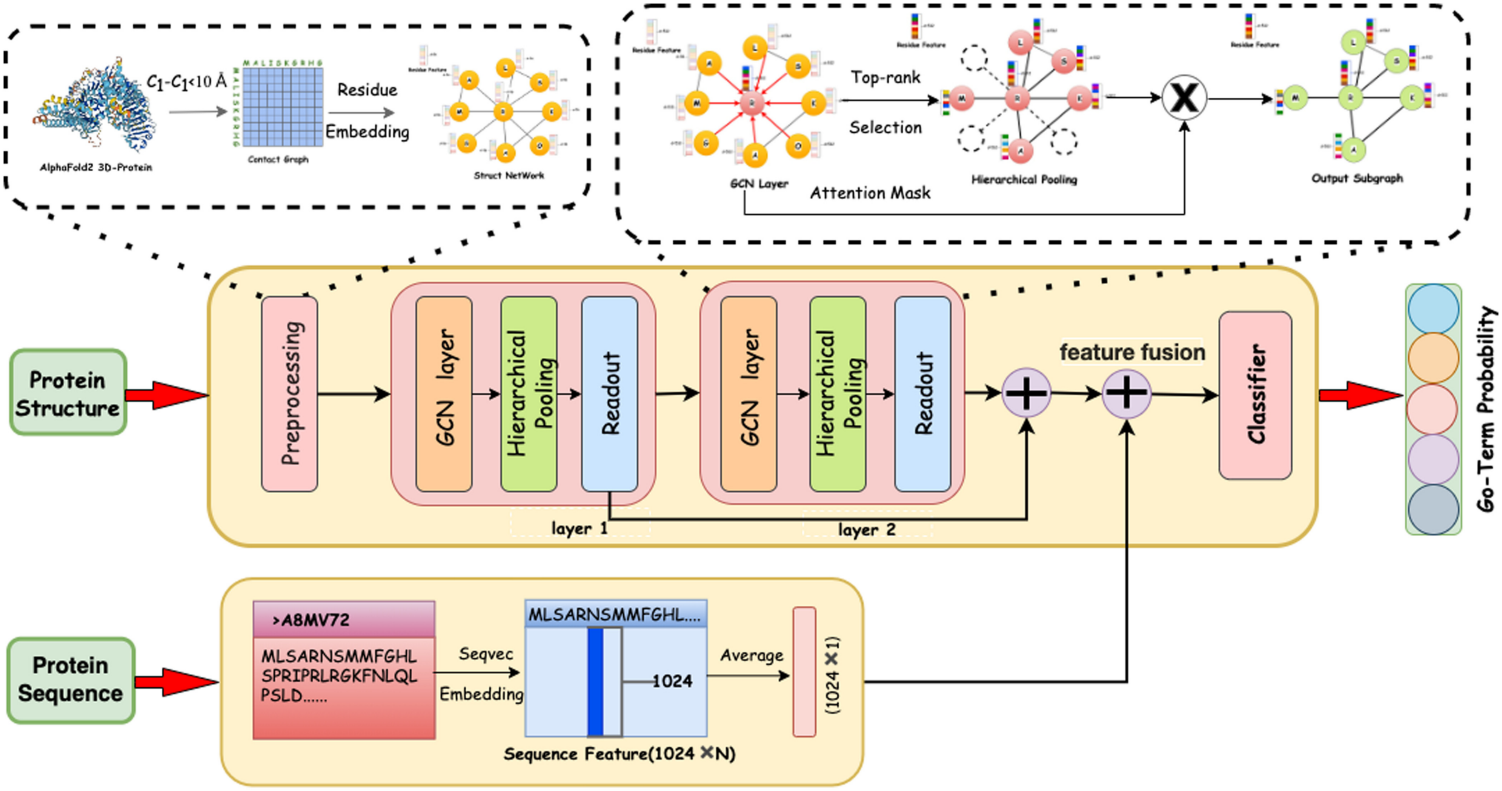
The Struct2Go model graph. The model’s input includes protein structure and protein sequence. In the preprocessing stage, the protein three-dimensional structure is transformed into a protein contact graph, and amino acid-level embedding is generated through Node2vec. At the same time, based on SeqVec, the protein sequence features are extracted and dimensionality is reduced to 1×1024. Then, through two layers of the self-attention graph pooling model, protein structure features are extracted, in which GCN aggregates neighbor information and generates node weights, Top-rank algorithm selects nodes according to weight values, and updates node features to generate subgraphs, and accumulates the feature values of the two readout layers as the output of protein structural features. Finally, the sequence and structure features of the protein are fused as the input of the classifier.

In this article, Struct2GO, a protein function prediction model that leverages multi-source data fusion, is proposed as shown as Fig. 1. Specifically, the model takes protein sequence information and protein structure information as inputs extracts sequence features through the SeqVec pre-training model and extracts structural features through the hierarchical graph pooling model based on the self-attention mechanism. To maximize the utilization of the protein structure information provided by AlphaFold2, the residue-level embedding is pre-trained in the protein structure network via Node2vec, which is then employed as the initial node feature of the pooling model. Numerous experiments have demonstrated that the protein function prediction model which combines structure and sequence can significantly enhance prediction accuracy. Simultaneously, the model eliminates the restrictions of the PPI network on feature extraction, thereby significantly improving the model’s generalizability.

## 2 Materials and methods

### 2.1 Datasets

In this experiment, we obtained human protein structure data predicted by AlphaFold2 from the EMBL-EBI database, including 23 391 protein structures. In this article, more than 560 000 data were screened from the gene ontology annotation labels corresponding to human proteins, and the annotations obtained by experiments, that is, evidence codes (Evidence Code) of “IDA,” “IPI,” “EXP,” “IGI,” “IMP,” “IEP,” “IC,” or “TA” were extracted, among which the human dataset included 20 395 data. Concurrently, we downloaded and parsed the most recent gene ontology data released by the official gene ontology website, construct the directed acyclic graph of gene ontology according to the terms of BPO, CCO, and MFO branches parsed, and complete the labels according to the above true path rules. It should be noted that most of the functional terms do not appear in the dataset or only annotate a few proteins, so this article filters out gene ontology nouns with a frequency lower than a certain threshold for each branch to reduce the sparsity of labels. After completion, the number of BPO, MFO, and CCO labels is 809, 273, and 298 respectively (see Supplementary Table S3).

### 2.2 Protein representation

#### 2.2.1 The construction of protein contact map

Protein structure and function are closely related. To better infer protein-related functions from protein structure information, we transform the three-dimensional protein structure into a two-dimensional protein contact map, construct a protein structure network to aggregate the information of adjacent residues, and finally obtain the protein structure features.

In terms of a specific implementation, we can obtain the three-dimensional atomic coordinates of the protein structure through AlphaFold2, and then calculate the relative distance between amino acid residues. If the Cα atom between them is less than 10 Å, it is considered that there is an edge directly connected between the two residues. We employed two distinct methods for generating contact maps: ANY-ANY and NBR, see Supplementary Fig. S1 for the relevant experimental results.

#### 2.2.2 Obtaining amino acid residue level features

In the protein structure network, each node is an amino acid residue. To obtain the features of the node, the most intuitive method is to use the one-hot encoding of 20 different amino acids, but this method cannot capture the position information of the same amino acid in different protein networks. Therefore, we utilize graph representation learning to acquire the structural information of the node in the protein network. Among the current algorithm, DeepWalk (Perozzi *et al.* 2014) is one of the most representative algorithms, which extends the word2vec (Mikolov *et al.* 2013) idea, and suppose that neighboring nodes have analogous embedding vectors. Node2vec (Grover and Leskovec 2016) is optimized through a biased random walk to acquire the successive vertices, i.e. given the current vertex *v*, the likelihood of visiting the subsequent vertex *x* is


#(1) Pci=xci-1=v=πvxZ ifv, x∈E0 otherwise,

where $\pi_{vx}$ is the unnormalized transition probability between vertex *v* and vertex *x*, and *Z* is the normalization constant. Node2vec introduces two hyperparameters *p* and *q* to regulate the random walk strategy. Assuming that the current random walk passes through (*t*, *v*) to reach vertex *v*, let$\;\pi_{vx}=\alpha_{pq}\left(t,x\right)\cdot w_{\mathrm{vx}}$,$w_{\mathrm{vx}}$ is the edge weight between vertex v and x.


#(2) αpqt,x=1p if dtx=01 if dtx=11q if dtx=2,

where $d_{tx}$ is the shortest path distance between vertex *t* and vertex *x*.

In terms of a specific implementation, this experiment is based on the open-source distributed machine learning platform Spark-On-Angel of Tencent and uses the efficient data storage, update, and sharing services provided by Spark to implement the node2vec algorithm for graph computing. In the proteins we input, the number of amino acid residues is below 1500, so we choose the length of the walk-in node2vec to be 30, *p* to be 0.8, *q* to be 1.2, combined with the one-hot encoding of each residue, and finally, we generate 1 × 50 dimensional feature vectors for each residue in the protein.

#### 2.2.3 Extraction of protein sequence features

In the natural language domain, there has been a rapid advancement of pre-trained models such as Bert (Devlin *et al.* 2018) and XLNet (Yang *et al.* 2019) in recent years, and many researchers have extended the models in the NLP field to the bio-sequence field, proposing a variety of pre-trained models for obtaining distributed representations of protein sequences, and the SeqVec model (Heinzinger *et al.* 2019) is widely employed among them. The SeqVec pre-trained model can extract semantic information related to function from the sequence and has achieved good results in tasks such as protein subcellular localization, secondary structure prediction, and functional prediction. Specifically, the SeqVec model uses the CharCNN (Zhang *et al.* 2015) algorithm to acquire local characteristics of amino acids, and then uses the BiLSTM algorithm to construct the language model. The single amino acid feature is obtained by averaging the field features and the language model. That is, for the kth amino acid, its representation is


#(3) SeqVeck=xkLM+hk,1LM+hk,2LM 

#(4) hk,jLM=h→k,jLM;h←k,jLM ,

where $x_{k}^{\text{LM}}$ is the 1024-dimensional character features output by the CharCNN layer, and ${\vec{h}}_{k,j}^{\text{LM}},\;{\overset{\leftarrow }{h}}_{k,j\;}^{\mathrm{LM}}$represents the 512-dimensional vectors output in a forward and backward of LSTM layers, respectively. These two output vectors are concatenated to form a 1024-dimensional feature $h_{k,j}^{\text{LM}}$ as the resultant of the *j*-layer BiLSTM model. Finally, the SeqVec model concatenates residue-level features into a 1024×N matrix and reduces the dimensionality of the matrix through principal component analysis or average aggregation to generate a 1×1024 matrix.

In terms of the specific implementation, this experiment uses the SeqVec model, which first pre-trains about 33M sequences in the UniRef50 database. Then the human protein sequences are taken as input. For each protein sequence, we can get a feature vector as the protein sequence feature, which is combined with its structural features in the subsequent model for downstream protein function prediction.

### 2.3 Model and implementation

Since the same protein may have multiple functions, the model is essentially a multi-label classification task. In this article, an attention-based graph pooling mechanism is adopted, which takes the above-obtained protein contact graph and amino acid residue features as input extract protein structural features through graph convolution and hierarchical pooling and integrates the above sequence features as the input of the downstream protein function prediction multi-label classifier. At the same time, the network layer and post-processing layer in the classifier ensure the hierarchical relationship between GO labels.

#### 2.3.1 Convolution layer

In this stage, we take the protein contact graph as the adjacency matrix and the amino acid residue features as the node feature in the graph and propagate its features between residues with similar structures and structures through graph convolution. We explored several widely used graph convolution functions, including Kipf and Welling graph convolutional layer (GraphConv) (Kipf and Welling 2016), Chebyshev spectral graph convolutions (ChebConv) (Defferrard *et al.* 2016), SAmpLe and aggregate convolutions (SAGEConv) (Hamilton *et al.* 2017), and Graph Attention (GAT) (Veličković *et al.* 2017). We compared the effects of different graph convolution methods on the results, and the experimental findings revealed that the two-layer GraphConv model attained the highest level of success. In each layer, a new hidden representation is obtained through neighbor message propagation and aggregation:


#(5) hl+1=σD˜-12AD˜-12hlΘ ,

where $h^{\left(l\right)}$ is the representation of the *l*th layer nodes, $\Theta \in R^{F\times F^{'}}\;$is the learnable convolutional weights, $\tilde{D}\in R^{N\times N^{'\;}}$is the $\tilde{A}\;$degree matrix, and $\tilde{A}\in R^{N\times N^{'}}$ is the adjacency matrix with self-connections.

#### 2.3.2 Self-attention graph hierarchical pooling layer

In recent years, the self-attention mechanism has been extensively employed in deep learning models, resulting in noteworthy outcomes, and allowing the model to focus more on significant features. Lee *et al.* (2019) introduced the self-attention method to the graph pooling model, and obtained importance scores of each node by stacking convolutional layers and transforming the output features into one-dimensional. Then we adopted the node selection algorithm proposed by Cangea *et al.* (2018), which retains some nodes and edges of the input graph and generates a new subgraph as the input of the next layer. The pooling ratio k determines the number of nodes that will be retained, and we select $\left\lceil k\left.N\right\rceil \right.$ nodes by the importance scores of each node obtained from the self-attention convolutional layer. In the application of the model, we use the two-head attention mechanism to obtain two importance scores for each node respectively and calculate the mean as the ultimate score. In the experiment, this method effectively improves the performance of the model.


#(6) idx=toprankZ,kN Zmask=Zidx,

#(7) Xout=X′⊙Zmask Aout=Aidx,idx,

where $X^{\prime }$ is the original feature of the retained node, $X_{\mathrm{out}}$ is the generated feature of the retained node, $Z_{\mathrm{mask}}$ is the importance score of the retained node, and $A_{\mathrm{out}}$ is the adjacency matrix of the subgraph generated by the retained node.

#### 2.3.3 Readout layer

Xu *et al.* (2018) proved in the paper that in the field of graph classification, compared with mean-pooling and max-pooling, sum-pooling shows better results. In sum-pooling, all node features in the graph are summed up, which can learn all labels and extract more information. In our hierarchical pooling model, we extract the graph features of this layer by splicing sum-pooling and max-pooling, and finally, sum the graph features of multiple layers as the structural features of the protein. The formula for each layer graph pooling is as follows:


#(8) S=1N∑i=1NXi||max⁡Xi ,

where *N* is the number of nodes in this layer, $X_{i}$ represents the feature of the *i*th node, and || represents the feature splicing.

## 3 Experiment and results

### 3.1 Experiment

To validate the effectiveness of Struct2Go, we divided the human protein dataset into training set, validation set, and test set in a ratio of 8:1:1 respectively to conduct experiments with three different prediction models. We compared the predicted results of the test set with those of the current mainstream models, including Naïve, BLAST, DeepGO, DeepGOA, DeepFRI, and GAT-GO. Naïve algorithm annotates GO terms according to the frequency, and BLAST is a protein sequence comparison technique that utilizes sequence similarity and dynamic programming to predict gene labels. DeepGO leverages both protein sequence information and PPI network data to infer gene ontology tags. DeepGOA innovatively introduces GCN to obtain knowledge guidance prediction in GO, DeepFRI transforms protein three-dimensional structure into a contact map and uses GCN to extract structural features for protein function prediction, and GAT-GO changes the aggregation function GCN to GAT based on DeepFRI and verifies it through experiments.

In this article, AUC, AUPR, and Fmax are selected as metrics to evaluate the accuracy of protein function prediction from different perspectives.

(The definition of specific parameters and formulas can be found in the Supplementary Data.) From Table 1, it is observable that our model has achieved a considerable enhancement in multiple metrics, which can be attributed to our processing and model design for the protein dataset when compared to other prevalent models. We fully mined the protein structure information provided by AlphaFold2 and combined it with the sequence feature method to achieve good results in protein function prediction. At the same time, we also see that in all branches, the MFO branch has good prediction results, while the BPO branch has lower accuracy, which may be related to the number of labels in different branches. For a fair evaluation of the model, metrics for all GO labels are provided and a histogram is plotted as shown in Supplementary Fig. S2. The metrics of the training set and test sets can be seen in Supplementary Table S4.

**Table 1.** Experimental results on human protein data.

Model	BPO			CCO			MFO		
	Fmax	AUC	AUPR	Fmax	AUC	AUPR	Fmax	AUC	AUPR
Naïve	0.347	0.501	0.568	0.571	0.477	0.372	0.336	0.498	0.532
BLAST	0.339	0.577	0.489	0.441	0.563	0.269	0.411	0.623	0.461
DeepGO	0.327	0.639	0.571	0.589	0.695	0.448	0.404	0.760	0.625
DeepGOA	0.385	0.698	0.622	0.629	0.757	0.500	0.477	0.820	0.710
DeepFRI	0.425	0.732	0.635	0.624	0.779	0.641	0.542	0.881	0.763
GAT-GO	0.462	0.586	0.512	0.647	0.831	0.681	0.633	0.912	0.776
Struct2GO	**0.481**	**0.873**	**0.661**	**0.658**	**0.942**	**0.763**	**0.701**	**0.969**	**0.796**

### 3.2 Ablation study

Then, we perform ablation experiments to assess the impact of each component in the Struct2GO model on the enhancement of performance, as shown in Table 2.

**Table 2.** Ablation experiment results on human protein data.

Methods	BPO			CCO			MFO		
	Fmax	AUC	AUPR	Fmax	AUC	AUPR	Fmax	AUC	AUPR
Without structure	0.361	0.788	0.427	0.544	0.886	0.680	0.422	0.863	0.634
Without one-hot	0.438	0.854	0.609	0.625	0.934	0.727	0.648	0.947	0.752
Without Node2vec	0.430	0.850	0.602	0.584	0.925	0.714	0.636	0.946	0.694
Without sequence	0.429	0.851	0.595	0.579	0.924	0.705	0.594	0.945	0.707
Struct2GO	**0.481**	**0.873**	**0.661**	**0.658**	**0.942**	**0.763**	**0.701**	**0.969**	**0.796**

The experiments involved extracting protein semantic features from individual sequences using the SeqVec pre-trained model, obtaining contact maps based on AlphaFold2’s atomic-level protein three-dimensional coordinates, and extracting protein structural features through hierarchical graph pooling. From the experimental data, it can be seen that the removal of any component will lead to the loss of model performance, which fully proves that all components of our model are effective. The ablation experiments reveal that, compared to protein semantic features obtained solely from single sequences, protein structural features have a significant impact on downstream function prediction tasks. Analogous to the findings of Arya *et al.* (2022), structural-based features are more effective in capturing amino acid mutations. Furthermore, our ablation experiment results also support the perspective of the conclusions drawn by Arya *et al.* (2022) indirectly.

### 3.3 Model analysis

We compare the different variants of each component in the model and verify through experiments that our model achieves the best results in each variant, as shown in Table 3. When extracting structural features from the protein contact graph, we use four different aggregation functions, GraphConv, ChebConv, GATConv, and SAGEConv, respectively. The experimental data reveal that the various aggregation functions have a minimal influence on the model performance, but GraphConv often achieves better results in all data. Next, we compare the effects of SumPool, AvgPool, and MaxPool on model performance when reading graphs. As Xu *et al.* (2018) stated, the SumPool method can accumulate more features and often achieve better results in tasks that distinguish graph structures. The Struct2GO model demonstrates that hierarchical graph pooling is more effective than global graph pooling, likely due to its capacity to efficiently extract pertinent information from protein contact graphs with a large number of nodes. Finally, we contrasted the outcomes of single-layer and double-layer self-attention layers when utilizing hierarchical pooling. The experimental results show that multi-head attention layers can learn more effective information and often perform better in experiments.

**Table 3.** Model comparison experiment results on human protein dataset.

Methods	BPO			CCO			MFO		
	Fmax	AUC	AUPR	Fmax	AUC	AUPR	Fmax	AUC	AUPR
Sturct2GO-GraphConv	**0.481**	**0.873**	**0.661**	**0.658**	**0.942**	**0.763**	**0.701**	**0.969**	**0.796**
Sturct2GO-ChebConv	0.465	0.868	0.745	0.637	0.938	0.719	0.665	0.952	0.695
Sturct2GO-GATConv	0.457	0.869	0.749	0.623	0.931	0.703	0.678	0.953	0.705
Sturct2GO-SAGEConv	0.471	0.868	0.735	0.642	0.937	0.713	0.683	0.955	0.702
Struct2GO-SumPool	**0.481**	**0.873**	**0.661**	**0.658**	**0.942**	**0.763**	**0.701**	**0.969**	**0.796**
Struct2GO-AvgPool	0.358	0.786	0.627	0.544	0.890	0.686	0.404	0.838	0.503
Struct2GO-MaxPool	0.457	0.864	0.731	0.633	0.936	0.720	0.667	0.953	0.696
Struct2GO-Hierarchical	**0.481**	**0.873**	**0.661**	**0.658**	**0.942**	**0.763**	**0.701**	**0.969**	**0.796**
Struct2GO-Global	0.364	0.789	0.613	0.542	0.890	0.683	0.402	0.838	0.601
Struct2GO-2_layer_attention	**0.481**	**0.873**	**0.661**	**0.658**	**0.942**	**0.763**	**0.701**	**0.969**	**0.796**
Struct2GO-1_layer_attention	**0.456**	**0.867**	**0.745**	**0.629**	**0.935**	**0.728**	**0.634**	**0.942**	**0.672**

### 3.4 Parameter sensitivity analysis

Then, we examined the effects of parameters such as dropout, learning rate, pooling ratio, and conv number on the model. We employed the control variable method, varying a single parameter at a time for multiple comparison experiments, and evaluated the actual impact of the parameter on the model performance by observing the performance of the model after training, to identify the optimal parameter value. The scope of hyperparameter comparison experiments is presented in Table 4.

**Table 4.** Range of hyperparameter comparison experiments.

Hyperparameter	Range
Dropout	0.3, 0.35, 0.4, 0.45, 0.5
Learning rate	0.1, 0.01, 0.001, 0.0001
Pooling ratio	0.25, 0.5, 0.75
Conv number	1, 2, 3, 4

**Figure 2.**
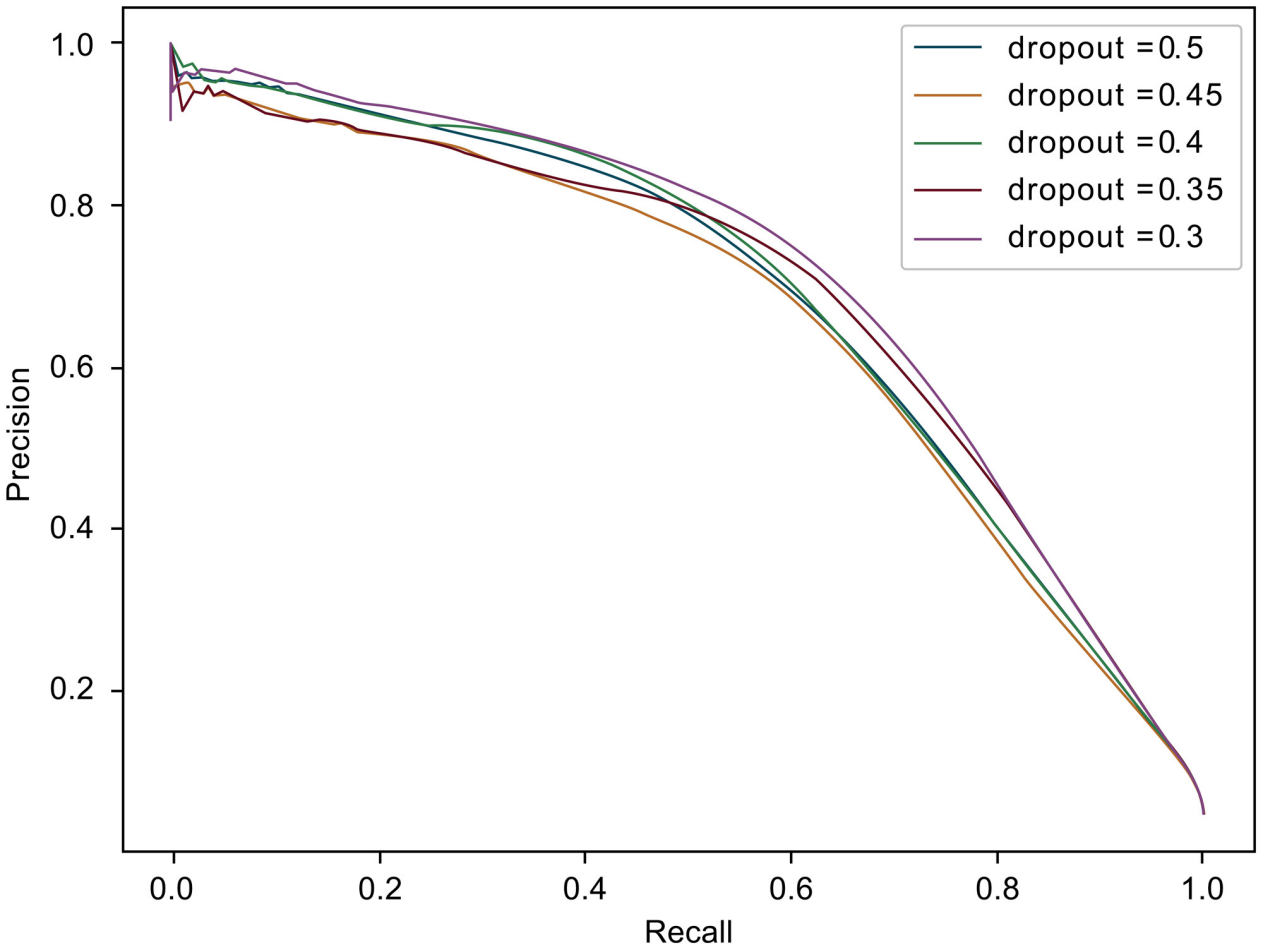
PR curve of Struct2GO with different dropout. The curve of different colors represents the influence of different dropout values on the performance of the model. By observing the PR graph, it can be found that the model shows the best performance and stability when the dropout value is 0.3.

The utilization of dropout in deep neural networks can mitigate overfitting and enhance the generalization capacity. From the experiments in Fig. 2, it is evident that varying dropouts have a negligible effect on the model performance. Among them, when the dropout is 0.3, the model achieves slightly better performance. Simultaneously, to expedite the convergence rate of the model, we opted for a more prudent dropout value of 0.3.

From the experimental data depicted in Supplementary Fig. S4, we observed that the model’s classification performance was weakest when the learning rate was 0.01, indicating that an excessively high learning rate could lead to the loss function fluctuating. When the learning rate decreased, the model’s convergence gradually improved, but this also necessitated a greater number of training cycles to reach the optimal value. Ultimately, taking into account both the number of training cycles and the model performance, we set the learning rate to 0.0001.

From the experimental data depicted in Supplementary Fig. S5, when the convolution number is 1, it means that we can only learn the features of the direct neighbors. As the convolution number increases, the nodes in the graph can learn more features of the indirect neighbors, but at the same time, it will also lead to the problem of overfitting. The experimental data reveal that the performance of various convolution numbers is only slightly dissimilar, and the model achieves the optimal performance when the convolution number is 2.

The pooled ratio represents the ratio of the number of nodes in the subgraph generated in the next layer in the hierarchical process to the original graph, that is, the pooling ratio. In Supplementary Fig. S6, if the pooling ratio is 1, it degenerates to global pooling. From the comparison of the experimental results in the graph, we find that the model performance is better when the pooling ratio is 0.75. When the pooling ratio is reduced to 0.25, the model performance has a significant decrease, which may be because the reduction of the number of nodes in the subgraph will affect the generalization ability of the model, so we set the pool ratio value to 0.75.

## 4 Conclusion

In this article, we propose a powerful end-to-end graph deep learning model Struct2Go, which can effectively and quickly annotate protein functions based on protein structure and sequence. Specifically, we adopt a graph pooling model to acquire structural features from the three-dimensional protein structure predicted by AlphaFold2 and integrate the sequence features extracted by Seqvec to train the protein function classifier. AlphaFold2 predicted three-dimensional protein structure data provides strong support for our functional prediction, which can enable us to abandon the constraints of PPI networks in previous works and effectively improve the generality of the model. At the same time, compared with the previous methods for predicting protein function based on experimentally determined protein structure, AlphaFold2 provides sufficient high-resolution structure information, which enables our model to perceive more homologous information and effectively improve the accuracy of prediction. The comparative experiments demonstrate that Struct2Go has attained the most advanced performance, thereby conclusively demonstrating the effective support of structural information for protein function prediction.

In our future work, we will continue to investigate novel methods and enhance the generality and precision of the Struct2Go model. In addition, the AlphaFold2 website provides us with 217 million protein structure datasets of multiple species, which can be used in future research to try large-scale cross-species protein function model training, which can effectively improve the generality of the model.

At the same time, in order to focus more on the influence of subtle structural changes on protein function prediction in future work, we can explore new approaches in protein structure feature extraction. For instance, we can investigate embedding the amino acid features extracted from sequence models into protein structural networks and explore novel random walk models to more comprehensively unearth valuable information within protein structures. In addition, we can also build a protein network based on structural similarity, with a single protein as the node, and use the effective information of homologous proteins in network propagation to improve the accuracy of the model prediction.

## Supplementary Material

btad637_Supplementary_Data

## References

[btad637-B1] Altschul SF , Gish W, Miller W et al Basic local alignment search tool. J Mol Biol 1990;215:403–10. 2231712 10.1016/S0022-2836(05)80360-2

[btad637-B2] Arya A , Mary Varghese D, Kumar Verma A et al Inadequacy of evolutionary profiles vis-a-vis single sequences in predicting transient DNA-binding sites in proteins. J Mol Biol 2022;434:167640. 35597551 10.1016/j.jmb.2022.167640

[btad637-B3] Brenner SE , Chothia C, Hubbard TJP et al Understanding protein structure: using scop for fold interpretation. Methods Enzymology 1996;266:635–43. 10.1016/s0076-6879(96)66039-x 8743710

[btad637-B4] Buchfink B , Xie C, Huson DH. Fast and sensitive protein alignment using DIAMOND. Nat Methods 2015;12:59–60. 25402007 10.1038/nmeth.3176

[btad637-B5] Cangea C , Veličković P, Jovanović N et al Towards sparse hierarchical graph *classifiers.* arXiv, arXiv:1811.01287, 2018, preprint: not peer reviewed.

[btad637-B6] Dawson NL , Lewis TE, Das S et al CATH: an expanded resource to predict protein function through structure and sequence. Nucleic Acids Res 2017;45:D289–95. 27899584 10.1093/nar/gkw1098 PMC5210570

[btad637-B7] Defferrard M , Bresson X, Vandergheynst P. Convolutional neural networks on graphs with fast localized spectral filtering. Adv Neural Inform. Process. Syst. 2016;29:3844–52.

[btad637-B8] Devlin J , Chang M W, Lee K et al Bert: pre-training of deep bidirectional transformers for language understanding. arXiv, arXiv:1810.04805, 2018, preprint: not peer reviewed.

[btad637-B9] Gligorijević V , Renfrew PD, Kosciolek T et al Structure-based protein function prediction using graph convolutional networks. Nat Commun 2021;12:3168. 34039967 10.1038/s41467-021-23303-9 PMC8155034

[btad637-B10] Grover A , Leskovec J. node2vec: Scalable Feature Learning for Networks. In: *Proceedings of the 22nd ACM SIGKDD International Conference on Knowledge Discovery and Data Mining*. San Francisco, CA: Association for Computing Machinery, 2016, 855–64. 10.1145/2939672.2939754 PMC5108654 27853626

[btad637-B11] Hamilton W , Ying Z, Leskovec J. Inductive representation learning on large graphs. Advn Neural Inform Process Syst 2017;30:1024–34.

[btad637-B12] Heinzinger M , Elnaggar A, Wang Y et al Modeling aspects of the language of life through transfer-learning protein sequences. BMC Bioinformatics 2019;20:723–17. 31847804 10.1186/s12859-019-3220-8 PMC6918593

[btad637-B13] Holm L , Sander C. Mapping the protein universe. Science 1996;273:595–603. 8662544 10.1126/science.273.5275.595

[btad637-B14] Jumper J , Evans R, Pritzel A et al Highly accurate protein structure prediction with AlphaFold. Nature 2021;596:583–9. 34265844 10.1038/s41586-021-03819-2 PMC8371605

[btad637-B15] Kipf TN , Welling M. Semi-supervised classification with graph convolutional networks. arXiv, arXiv:1609.02907, 2016, preprint: not peer reviewed.

[btad637-B16] Krissinel E. On the relationship between sequence and structure similarities in proteomics. Bioinformatics 2007;23:717–23. 17242029 10.1093/bioinformatics/btm006

[btad637-B17] Kulmanov M , Hoehndorf R. DeepGOPlus: improved protein function prediction from sequence. Bioinformatics 2020;36:422–9. 31350877 10.1093/bioinformatics/btz595 PMC9883727

[btad637-B18] Kulmanov M , Khan MA, Hoehndorf R et al DeepGO: predicting protein functions from sequence and interactions using a deep ontology-aware classifier. Bioinformatics 2018;34:660–8. 29028931 10.1093/bioinformatics/btx624 PMC5860606

[btad637-B19] Lan L , Djuric N, Guo Y et al MS-kNN: protein function prediction by integrating multiple data sources. BMC Bioinformatics 2013;14(Suppl. 3):S8. 10.1186/1471-2105-14-S3-S8 PMC3584913 23514608

[btad637-B20] Lee J , Lee I, Kang J. Self-Attention graph pooling. In: Kamalika C, Ruslan S (eds), Proceedings of the 36th International Conference on Machine Learning. Long Beach, CA, US: Proceedings of Machine Learning Research (PMLR); 2019. pp. 3734–43.

[btad637-B21] Mikolov T , Chen K, Corrado G et al Efficient estimation of word representations in vector space. arXiv, arXiv:1301.3781, 2013, preprint: not peer reviewed.

[btad637-B22] Mitchell AL , Attwood TK, Babbitt PC et al InterPro in 2019: improving coverage, classification and access to protein sequence annotations. Nucleic Acids Res 2019;47:D351–60. 30398656 10.1093/nar/gky1100 PMC6323941

[btad637-B23] Perozzi B , Al-Rfou R, Skiena S. DeepWalk: online learning of social representations. In: *Proceedings of the 20th ACM SIGKDD International Conference on Knowledge Discovery and Data Mining*. New York: Association for Computing Machinery, 2014, 701–10.

[btad637-B24] Sebastian A , Contreras-Moreira B. The twilight zone of cis element alignments. Nucleic Acids Res 2013;41:1438–49. 23268451 10.1093/nar/gks1301 PMC3561995

[btad637-B25] The Gene Ontology Consortium. Expansion of the gene ontology knowledgebase and resources. Nucleic Acids Res 2017;45:D331–8. 27899567 10.1093/nar/gkw1108 PMC5210579

[btad637-B26] UniProt Consortium. UniProt: a worldwide hub of protein knowledge. Nucleic Acids Res 2018;47:D506–15. 10.1093/nar/gky1049 PMC6323992 30395287

[btad637-B27] Veličković P , Cucurull G, Casanova A et al Graph attention networks. arXiv, arXiv:1710.10903, 2017, preprint: not peer reviewed.

[btad637-B28] Wu K , Wang L, Liu B et al PSPGO: Cross-species heterogeneous network propagation for protein function prediction. IEEE/ACM Trans Comput Biol Bioinform 2023;20:1713–24. 36251905 10.1109/TCBB.2022.3215257

[btad637-B29] Xu K , Hu W, Leskovec J et al How powerful are graph neural networks? arXiv, arXiv:1810.00826, 2018, preprint: not peer reviewed.

[btad637-B30] Yang Z , Dai Z, Yang Y et al Xlnet: generalized autoregressive pretraining for language understanding. Adv. Neural Inform. Process. Systems 2019;32:5753–63.

[btad637-B31] You R , Yao S, Mamitsuka H et al DeepGraphGO: graph neural network for large-scale, multispecies protein function prediction. Bioinformatics 2021;37:i262–71. 34252926 10.1093/bioinformatics/btab270 PMC8294856

[btad637-B32] Zhang X , Zhao J, LeCun Y. Character-level convolutional networks for text classification. In: *Proceedings of the 28th International Conference on Neural Information Processing Systems—Volume 1*. Montreal, Canada: MIT Press, 2015, 649–57.

