# Supplementary material for: Struct2GO: protein function prediction based on graph pooling algorithm and AlphaFold2 structure information
Source: Bioinformatics. 2023 Oct 17;39(10):btad637. doi: 10.1093/bioinformatics/btad637 (PMC10612405; doi:10.1093/bioinformatics/btad637)
Supplement: btad637_Supplementary_Data [file btad637_supplementary_data.pdf]

Supplemental Data

Evaluation metrics

AUC, AUPR and Fmax are selected as metrics to evaluate the accuracy of protein function prediction from different perspectives.

(1) AUC first calculates the Receiver Operation Curve (ROC curve) on each functional label. The curve is constructed by connecting points on the coordinate axis corresponding to the true positive rate (True Positive Rate) and false positive rate (False Positive Rate) at different thresholds. The coordinate axis is the true positive rate as the y-axis and the false positive rate as the x-axis. Then, the area of the coverage area formed by the curve and the x-axis is taken as the AUC value of the functional label, and the average result of these AUC values can reflect the discrimination ability of the model.

(2) AUPR is the area under the precision-recall curve (PR curve), which is the relationship between the precision rate of the classification model at different thresholds and the recall rate, reflecting the classification ability of the model at different discrimination thresholds.

(3) Fmax takes into account the precision and recall of the model. For a given threshold, the average precision (pr) and average recall (rc) on the test set, and Fmax are defined as follows:

$$pr(\tau) = \frac{1}{m(\tau)} \sum_{i=1}^{m(\tau)} pr_i(\tau)$$

$$rc(\tau) = \frac{1}{n} \sum_{i=1}^{n} rc_i(\tau)$$

$$Fmax(\tau) = \max_{\tau \in [0,1]} \left\{ \frac{2 \cdot pr(\tau) \cdot rc(\tau)}{pr(\tau) + rc(\tau)} \right\}$$

Where $m(\tau)$ refers to the number of proteins whose prediction probability of at least one functional label is greater than or equal to the threshold τ, n and refers to the total number of proteins in the test set. The precision $pr_i(\tau)$ and $rc_i(\tau)$ recall of the i-th protein under the threshold τ are defined as follows:

$$pr_i(\tau) = \frac{|T \cap P_i(\tau)|}{|P_i(\tau)|}$$

$$rc_i(\tau) = \frac{|T \cap P_i(\tau)|}{|T|} \quad (13)$$

Where T represents the set of functional labels, $P_i(\tau)$ refers to the set of predicted labels of the i-th protein under the threshold τ, and the symbol $|\cdot|$ represents the size of the set.

Supplementary Figures 1: we augmented our research with two distinct methodologies for generating protein contact maps as comparative analyses.

1) ANY-ANY: If any of the two atoms which from different amino acids distance <10 Å, there will have an edge.

2) NBR: If the distance between their Rosetta neighbor atoms is less than sum of the neighbor radii of the amino acid pair, there will have an edge. Rosetta neighbor atoms are defined as Cβ atom for all amino acids except glycine where Cα is used.

We train different methods for generating protein contact maps on proteins with experimental MF-GO annotations.

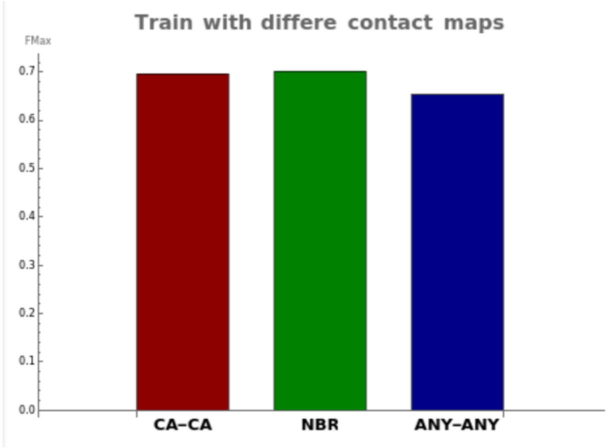

Comparison of Experimental Results for Three Different Protein Contact Map Generation Methods

Based on the experimental data, we can observe the following findings:

1) Generating contact maps using the ANY-ANY method yields suboptimal results. Setting the 10Å distance for any-any atom may result in many atoms being considered in contact, losing the ability to effectively filter meaningful interactions.

2) The NBR method shows a slight improvement, possibly because this approach more directly considers the relationships between amino acid side chains.

In our experiments, we computed protein contact maps using three different methods: CA-CA, NBR, and ANY-ANY, all with a threshold set at 10Å. From the experimental results, it is evident that ANY-ANY performed the worst. DeepFRI (Gligorijević, et al., 2021) have experimented with different distance thresholds for CA-CA and ANY-ANY contact map. The conclusion is that a threshold of 10Å works better for CA-CA, while a threshold of 6.5 is more effective for ANY-ANY. And they also found that these methods produced similar results when trained on contact map with CA-CA distance of 10Å, producing slightly better results. Therefore, based on the findings in DeepFRI (Gligorijević, et al., 2021), we ultimately chose the CA-CA method with a distance threshold set to 10Å to generate the contact maps.

Supplementary Figures 2: We acquired performance metrics for all GO labels in the test sets of MF, CC, and BP, and plotted their statistical histograms as follows:

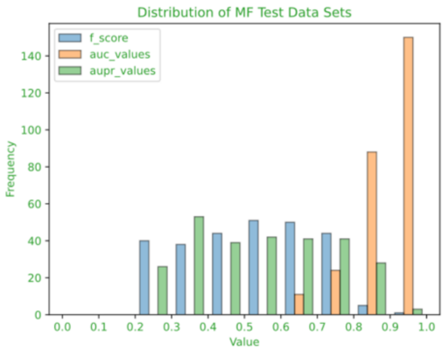

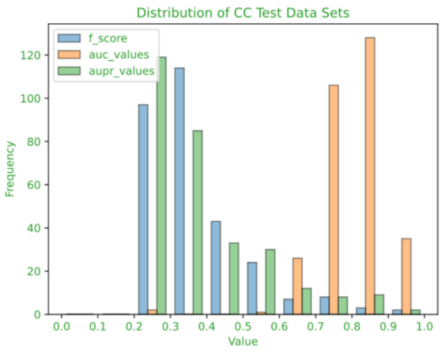

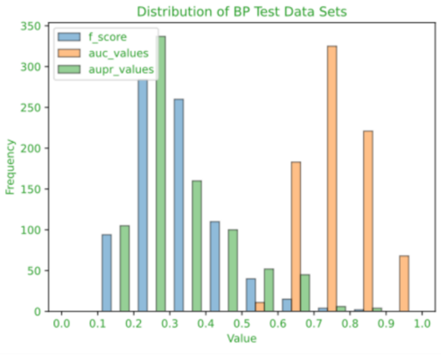

Supplementary Figures 3: we provide GO terms corresponding to all human proteins in the BP, MF, and CC branches, and used histograms to count the number of GO terms for different proteins.

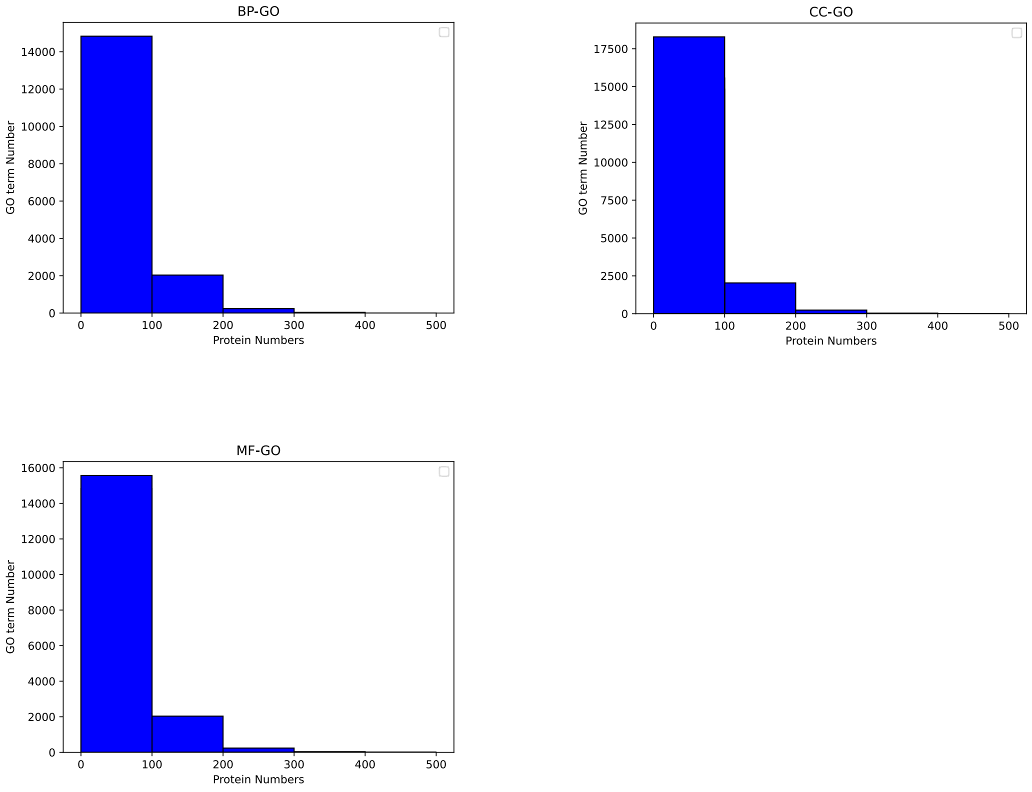

Supplementary Figures 4: The curves of different colors represent the influence of different learning rates on the convergence of the model. We weigh the performance of the model and the training time, and finally select the lr value as 0.0001.

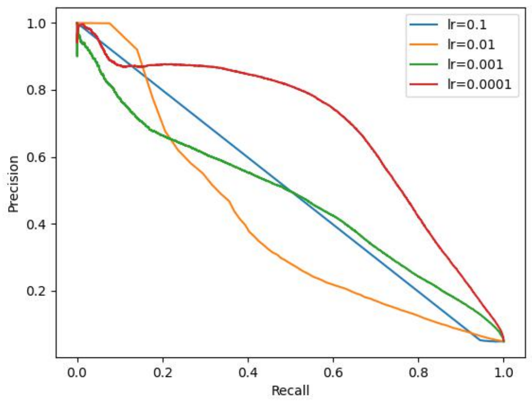

PR curve of Struct2GO with different learning rate.

Supplementary Figures 5: The curves of different colors represent the impact of different convolution numbers on the performance of the model. The difference between different values is slightly dissimilar and larger values can easily lead to overfitting problems, so we finally choose the value of num_convs to be 2.

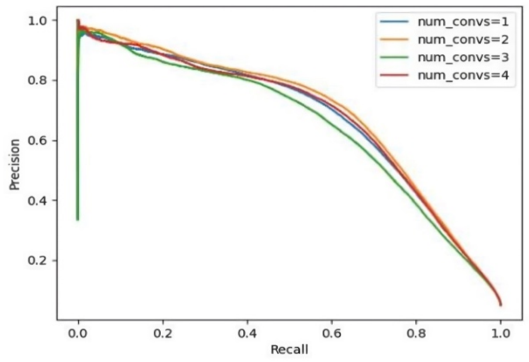

PR curve of Struct2GO with different conv number.

Supplementary Figures 6: It represents the ratio of the number of nodes in the subgraph generated in the next layer in the hierarchical process. According to the PR curve, we choose the value of pooling ratio is 0.75.

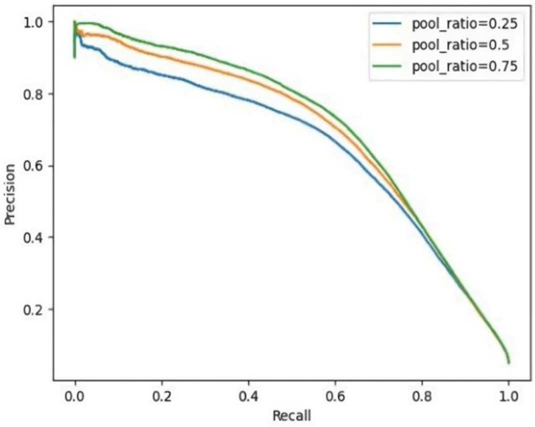

PR curve of Struct2GO with different pooling ratio.

Supplementary Table1: To ensure the relationship between parent and child nodes in predicting GO labels, comparative experimental results are presented after adding a Network Layer and a Post-processing Layer to the previous model.

Model	BPO			CCO			MFO		
	Fmax	AUC	AUPR	Fmax	AUC	AUPR	Fmax	AUC	AUPR
Previous model	**0.475**	**0.870**	**0.652**	**0.653**	**0.938**	**0.735**	**0.696**	**0.967**	**0.782**
Modified Model	**0.481**	**0.873**	**0.661**	**0.658**	**0.942**	**0.763**	**0.701**	**0.969**	**0.796**

Supplementary Table 2: The following table shows what source data information is provided in Source Data and the description of the corresponding files.

File/Folder name	Description
predicted_struct_protein_data	Alphafold2 predicted human protein 3D structure datasets.
protein_contact_map	Computed CA-CA protein contact map.
struct_feature	Protein structural features.
dict_sequence_feature	Protein sequence features.
gos_bp.csv	GO terms corresponding to all human proteins in the BP branch.
gos_mf.csv	GO terms corresponding to all human proteins in the MF branch.
gos_cc.csv	GO terms corresponding to all human proteins in the CC branch.

Supplementary Table 3: We calculate the proportion of filtered labels in our experiments relative to the actual number of real labels. We have organized the aforementioned data as shown in the table below.

Branch	Filtered CO-Terms	Actual GO-Terms	Percent
BP	809	3207	25.23%
MF	273	1810	15.08%
CC	298	1879	15.87%

Supplementary Table 4: The metrics of the model on the training set, validation set, and test set are summarized in the table below.

Methods	BPO			CCO			MFO		
	**Fmax**	**AUC**	**AUPR**	**Fmax**	**AUC**	**AUPR**	**Fmax**	**AUC**	**AUPR**
Train Sets	**0.491**	**0.883**	**0.681**	**0.671**	**0.955**	**0.778**	**0.715**	**0.971**	**0.799**
Valid Sets	**0.485**	**0.881**	**0.673**	**0.660**	**0.951**	**0.774**	**0.707**	**0.971**	**0.796**
Test Sets	**0.481**	**0.873**	**0.661**	**0.658**	**0.942**	**0.763**	**0.69**	**0.967**	**0.782**
